# Rapid 3D bioprinting of a multicellular model recapitulating pterygium microenvironment

**DOI:** 10.1016/j.biomaterials.2022.121391

**Published:** 2022-01-28

**Authors:** Zheng Zhong, Jing Wang, Jing Tian, Xiaoqian Deng, Alis Balayan, Yazhi Sun, Yi Xiang, Jiaao Guan, Jacob Schimelman, Henry Hwang, Shangting You, Xiaokang Wu, Chao Ma, Xiaoao Shi, Emmie Yao, Sophie X. Deng, Shaochen Chen

**Affiliations:** a Department of NanoEngineering, University of California San Diego, La Jolla, CA, 92093, USA; b Department of Human Genetics, David Geffen School of Medicine, University of California Los Angeles, Los Angeles, CA, 90095, USA; c School of Medicine, University of California San Diego, La Jolla, CA, 92093, USA; d Stein Eye Institute, University of California Los Angeles, Los Angeles, CA, 90095, USA

**Keywords:** 3D bioprinting, Pterygium, Epithelial mesenchymal transition, Stem cells, Hydrogels, Disease model, Tissue engineering

## Abstract

Pterygium is an ocular surface disorder with high prevalence that can lead to vision impairment. As a pathological outgrowth of conjunctiva, pterygium involves neovascularization and chronic inflammation. Here, we developed a 3D multicellular *in vitro* pterygium model using a digital light processing (DLP)-based 3D bioprinting platform with human conjunctival stem cells (hCjSCs). A novel feeder-free culture system was adopted and efficiently expanded the primary hCjSCs with homogeneity, stemness and differentiation potency. The DLP-based 3D bioprinting method was able to fabricate hydrogel scaffolds that support the viability and biological integrity of the encapsulated hCjSCs. The bioprinted 3D pterygium model consisted of hCjSCs, immune cells, and vascular cells to recapitulate the disease microenvironment. Transcriptomic analysis using RNA sequencing (RNA-seq) identified a distinct profile correlated to inflammation response, angiogenesis, and epithelial mesenchymal transition in the bioprinted 3D pterygium model. In addition, the pterygium signatures and disease relevance of the bioprinted model were validated with the public RNA-seq data from patient-derived pterygium tissues. By integrating the stem cell technology with 3D bioprinting, this is the first reported 3D *in vitro* disease model for pterygium that can be utilized for future studies towards personalized medicine and drug screening.

## 1. Introduction

As an essential part of the ocular surface, conjunctiva is a mucosal stratified epithelial membrane that covers the major surface of sclera and provides functions for lubrication, mechanical support, and immune responses [1,2]. The conjunctival epithelium contains goblet cells producing mucins that comprise the tear film, which is a dynamic fluidic layer critical for the homeostasis of the ocular surface [3,4]. The inflammation and damage of conjunctiva caused by disease or injury could lead to a variety of symptoms, including dry eye and visual impairment [5,6]. Despite the high prevalence, the pathogenetic mechanism for many of the conjunctival diseases are unclear [5,6]. Pterygium is a pathological overgrowth of vascularized conjunctiva that invade the cornea across the limbus and compromise vision [7,8]. With little pharmaceutical treatments reported, patients with severe pterygium often require surgical interventions to restore basic visual function, but the prevention of post-surgical recurrence can be challenging [9] [–] [11]. Pterygium is a dynamic ecosystem orchestrated by multiple cell types, including stem cells, with chronic inflammation and angiogenesis being the two major hallmarks [8,12] [–] [16]. To study the pathogenesis and drug testing for pterygium, various types of disease models, including animal models and *in vitro* cultured cells, have been developed but are beset by the reproducibility, limited scalability, and the lack of heterogeneity on cell population, which contributed to the pathologically relevant cellular interaction [17] [–] [21]. With the recent technological advances, 3D engineered models with the capacity to recapitulate the multicellular microenvironment and support high-throughput drug screening have become a promising solution for pterygium disease modeling [22].

In the past decade, with the development of stem cell technologies, disease modeling based on tissue engineering of human stem cells has been widely explored to develop clinically relevant patient-specific models to replace small animal models for personalized medicine [23, 24]. 3D bioprinting is an emerging technology for the fabrication of functional 3D tissue structures with tailored biological and mechanical properties [25-28]. Among different 3D bioprinting techniques, digital light processing (DLP)-based 3D bioprinting method stands out because of its rapid fabrication speed, fine resolution at a microscale scale, and high cell integrity post-fabrication [25,29]. DLP-based bioprinting has been utilized to fabricate synthetic tissues for disease modeling of multiple organs and tissues, including heart, liver, brain, alveoli, spinal cord and bone [30] [–] [35]. Conjunctival stem cells (CjSCs) are bipotent stem cells that can give rise to both conjunctival goblet cells and conjunctival keratinocytes, and thereby hold tremendous potential in modeling the conjunctival microenvironment [4,36] [–] [38]. However, the lack of knowledge of both their microenvironment and a viable *in vitro* expansion method has limited the applications of CjSCs in tissue engineering [39] [–] [43]. We have previously reported DLP-based 3D bioprinting of microscale hydrogel constructs encapsulating rabbit CjSCs with the stem cell properties and differentiation potency preserved [44].

In this study, we explored the DLP-based 3D bioprinting method for primary hCjSCs and developed a bioprinted multicellular pterygium model. We first harvested the hCjSCs from donor tissues and expanded them with a feeder-free *in vitro* culture system. Using a customized DLP-based 3D bioprinter, we printed hydrogel scaffolds that were able to support the viability, stemness, and differentiation potency of encapsulated hCjSCs. Next, we performed multicellular bioprinting that combined hCjSCs along with immune cells and vasculature to develop a bioprinted 3D pterygium model. The bioprinted pterygium model was then subjected to global transcriptomic analysis to in-depth characterize the disease phenotypes. Furthermore, we validated our bioprinted model with published datasets of patient-derived pterygium tissues. The cellular interactions and signaling pathways revealed from the multicellular bioprinted model provide a greater understanding of pterygium pathogenesis. To the best of our knowledge, this is the first report of a 3D *in vitro* disease model mimicking the multicellular microenvironment of pterygium. The DLP-based 3D bioprinting of hCjSCs developed here can be translatable for clinical use in personalized medicine.

## 2. Materials and methods

### 2.1. Primary cell isolation, cell culture and cell doubling quantification

Fresh corneoscleral tissues were provided by One Legacy or Saving Sight Eye Bank with the consent for research use (Supplementary Table 4). The human corneoscleral tissue handling protocol has been evaluated and exempted by the University of California, Los Angeles (UCLA) Institutional Review Boards (IRB#12–000363). All experimental work adhered to the tenets of the Declaration of Helsinki, and the overall procedure was approved by the University of California, San Diego Institutional Biosafety Committee. Primary human conjunctival epithelial cells were isolated from the bulbar conjunctiva on the scleral surface that was 2–4 mm away from the limbus. Dissected tissues were subjected to mincing and a 30–60-min digestion with 0.5% type IV collagenase (Sigma Aldrich) at 37 °C under agitation. Following the collagen digestion, the cells were further digested with 0.25% trypsin-EDTA (ThermoFisher Scientific).

The isolated hCjSCs were cultured on a collagen I (ThermoFisher Scientific) surface as previously described [44]. The epithelial cell culture medium was made with Dulbecco’s Modified Eagle Medium (DMEM)/F-12 (3:1) supplemented with 10% (v/v) fetal bovine serum (FBS, ThermoFisher Scientific), 1% (v/v) penicillin-streptomycin (ThermoFisher Scientific), 1% (v/v) insulin-transferrin-selenium (ThermoFisher Scientific), 400 ng/ml hydrocortisone (Sigma Aldrich), 0.1 nM cholera toxin (Sigma Aldrich), 10 ng/ml recombinant human epidermal growth factor (EGF, R&D System), and 2 nM reverse T3 (Sigma Aldrich). The conjunctival stem cell culture medium (CjSCM) was made by adding 10 μM ROCK inhibitor Y27632 (Tocris Bioscience), 1 μM A83-01 (STEMCELL Technologies), and 1 μM DMH1 (STEMCELL Technologies), and used for the hCjSCs culture. Conjunctival goblet cell differentiation was performed using Keratinocyte SFM (ThermoFisher Scientific) supplemented with bovine pituitary extract (BPE), 10 ng/ml recombinant KGF (Biolegend), 10 ng/ml recombinant EGF (Biolegend), 1% (v/v) P–S, 10 ng/ml recombinant BMP4 (R&D System), and 100 ng/ml IL13 (Biolegend) [43-45]. M2 macrophages were acquired by differentiating THP-1 monocytes (ATCC). THP-1 cells were maintained with RPMI1640 medium (ThermoFisher Scientific) with 10% (v/v) FBS, and M2 differentiation was done by incubating the THP-1 cells in 200 ng/ml tetradecanoyl phorbol acetate (PMA, Sigma Aldrich) for 48 h, followed by incubation in complete RPMI 1640 medium for 24 h, and then in 20 ng/ml interleukin 4 (IL4, Biolegend) and 20 ng/ml interleukin 13 (IL13, Biolegend) for another 48 h. Human umbilical vein endothelial cells (HUVECs, Lonza) were cultured with Endothelial Cell Growth Medium-2 (EGM^™^-2, Lonza). C3H/10T1/2s mouse embryonic fibroblasts (10T1/2s, ATCC) were cultured with DMEM with 10% (v/v) FBS. For the cell culture of the 3D bioprinted pterygium model and the corresponding 2D control, complete EGM^™^-2 was mixed 1:1 with the epithelial cell culture medium and supplemented with 10 μM Y27632.

To quantify cell doubling, pre-cultured cells isolated from primary conjunctival epithelium were seeded on collagen I coated 12-well plate (Corning) with 40,000 cells per well. The epithelial cell culture medium was used as the control medium, and the cells were then cultured with either CjSCM or control medium. The cells were passaged when the confluence reached 90% and the cell count was measured manually every time with a hemocytometer (Fisher Scientific). The cells (100,000 cells per well) were seeded on collagen I coated 6-well plate (Corning) for the next round and the process was repeated. The cell doubling time (DT) was calculated as: *DT = ΔT·ln* 2/*ln*(*Q*2/*Q*1). *ΔT*: culture time. *Q1, Q2*: the number of cells at the beginning and at the end.

### 2.2. Material synthesis

The materials for bioprinting, gelatin methacryloyl (GelMA) and hyaluronic acid glycidyl methacrylate (HAGM), were prepared as previously described [44,46] [–] [48]. For the synthesis of GelMA, type A porcine skin gelatin (Sigma Aldrich) was dissolved in a 0.25 M carbonate-bicarbonate (3:7) solution (pH 9) to make a 10% (w/v) solution. Then, methacrylic anhydride (Sigma Aldrich) was added dropwise, followed by 1-h reaction at 50 °C with constant stirring. The products were dialyzed using 13.5 kDa dialysis membranes (Repligen), lyophilized, and stored at −80 °C. The synthesized GelMA had an approximate degree of methacrylation of 95% [47]. For HAGM, 1% (w/v) hyaluronic acid solution was made by dissolving sodium hyaluronate (Lifecore Biomedical) in water: acetone (1:1) solution with continuous stirring in dark at room temperature and incubated overnight. Next, triethylamine (Sigma Aldrich) was slowly added in the reaction and mixed thoroughly, then glycidyl methacrylate (GM, Sigma Aldrich) was also added dropwise, and reacted overnight at room temperature with Argon seal and constant stirring, followed by acetone precipitation. The products were collected with vacuum filtration, dissolved again with DI water, dialyzed, lyophilized, and stored at −80 °C. The resultant HAGM had an approximate degree of methacrylation of 35% [46].

The photoinitiator lithium phenyl-2,4,6-trimethylbenzoylpho sphinate (LAP) was synthesized following previous publication [44, 46]. Briefly, dimethyl phenylphosphonite (Sigma Aldrich) was added dropwise to an equimolar amount of 2,4,6-trimethylbenzoyl chloride (Acros Organics), and reacted for 18 h at room temperature with constant stirring. Then, a solution of lithium bromide (Sigma Aldrich) in 2-butanone (Sigma Aldrich) was added into the reaction, and incubated overnight at room temperature, followed by filter-washing with 2-butanone. The resultant solidified LAP was made into powder and stored in the dark at 4 °C.

### 2.3. DLP-based 3D bioprinting

A customized DLP-based 3D bioprinting system was built with projection optics, a 365 nm light source (Hamamatsu), a motion controller (Newport), and a digital micromirror device (DMD, Texas Instruments). The digital patterns were generated with MATLAB and inputted to the DMD chip through a custom-built coordination software. The thickness of the printed structures was controlled by a spacer made of polydimethylsiloxane (PDMS). The bioprinted hydrogel structures were printed on methacrylated coverslips upon light exposure, then rinsed with warm DPBS before subjected to culture in 5% CO_2_ at 37 °C. For the multilayered printing, the bioink was loaded on a PDMS base and the thickness of the structure was controlled by the motion controller. After printing the first layer, the excess uncrosslinked material was washed off with warm DPBS before the bioink of the second layer was loaded.

The pre-polymer solution for the printing was made by dissolving GelMA, HAGM, and LAP with DPBS (ThermoFisher Scientific) and filtered with a 0.22 μm syringe filter (Millipore Sigma). 5% or 8% (w/v) GelMA with 0.25% (w/v) LAP, and 2.5% (w/v) GelMA with 1% (w/v) HAGM and 0.25% (w/v) LAP were made accordingly. The 5% (w/v) GelMA was used as the soft condition for hCjSCs bioprinting while the 8% (w/v) GelMA was used as the stiff condition. 2.5% (w/v) GelMA with 1% (w/v) HAGM was used for the bioprinting of HUVECs and 10T1/2s. Before printing, the cells were digested, filtered with 70 μm cell strainers (Corning), quantified for the cell concentration, and pelleted with desired quantity. For hCjSCs bioprinting, the bioink contained 2 × 10^7^ cells/mL of hCjSCs. For multilayered printing of 3D pterygium model, the stem cell layer contained 2 × 10^7^ cells/mL of hCjSCs plus 1 × 10^7^ cells/mL of macrophages while the vascular layer contained 2 × 10^7^ cells/mL of HUVECs and 4 × 10^5^ cells/mL of 10T1/2s (50:1). For the 3D control, the scaffolds were fabricated with 5% GelMA with 2 × 107 cells/mL hCjSCs.

### 2.4. Mechanical characterization

The compressive Young’s modulus was measured using MicroTester (CellScale) following manufacturer’s instructions. GelMA cylinders with 500 μm-diameter and 500 μm-thickness were printed and incubated overnight in DPBS at 37 °C. Briefly, two cycles of predetermined compression were done to remove the hysteresis of the samples. Then, the samples were compressed by 10% strain with a rate of 2 μm/s while the force and displacement were recorded. The data was then processed with a custom-made MATLAB script.

### 2.5. Immunoassays and flow cytometry

For the immunofluorescence staining of 2D cultured cells, cells grown on Millicell EZ slides (Millipore Sigma) were washed twice with DPBS and fixed with 4% (w/v) paraformaldehyde (PFA, FUJIFILM Wako). The fixed samples were permeabilized and blocked with 5% bovine serum albumin (BSA, Sigma Aldrich) with 0.3% Triton X-100 (Sigma Aldrich) and 0.1% TWEEN^®^ 20 (Sigma Aldrich) for 1 h at room temperature. For the staining of mucin, samples were permeabilized with 0.2% Triton X-100 in DPBS for 10 min, following by 1-h blocking with 5% BSA. Then, the samples were incubated with primary antibody solution overnight. The secondary antibody with different conjugated fluorophores (Alexa Fluor^®^, Cell Signaling Technology) were diluted with 5% BSA and incubated with the samples for 1 h at room temperature. The antibody information was enclosed in Supplementary Table 1. The samples were stained with 4′,6-Diamidino-2-Phenylindole (DAPI, ThermoFisher Scientific) for the nuclear illustration and mounted with Fluoromount-G^™^ Mounting Medium (ThermoFisher Scientific). To stain the bioprinted samples, samples were fixed and stained following the same procedures, expect the last step of mounting.

For flow cytometry, encapsulated cells were released from the bioprinted scaffolds by enzymatical digestion with collagenase IV. The released cells were further digested with 0.25% trypsin-EDTA and filtered with a 70 μm cell strainer to obtain single cell suspension. The samples were then subjected to direct staining or fixed with Cytofix^™^ Fixation Buffer (BD). For immunostaining, fixed cells were permeabilized with 0.2% Triton X-100 in Cell Staining Buffer (Biolegend) for 2 min, and then incubated for 20 min with the diluted primary antibody, and secondary antibody, respectively. The cells were washed with Cell Staining Buffer between each step. BD Accuri^™^ C6 flow cytometer was used in the experiment and the resultant data was processed using FlowJo.

### 2.6. Viability tests

The viability of the encapsulated cells was evaluated using flow cytometry with propidium iodide (PI, Biolegend) staining and the LIVE/DEAD^®^ viability/cytotoxicity kit (ThermoFisher Scientific). For PI staining, samples were incubated with a diluted PI solution (10 μl per million cells in 0.5 ml/test) for 15 min at 4 °C before analysis. For the LIVE/DEAD^®^ staining, samples were incubated with 2 μM calcein acetoxymethyl ester and 4 μM ethidium homodimer diluted in DPBS, for 30 min at 37 °C, followed by imaging.

### 2.7. RNA extraction and real time qPCR

RNA was isolated with a method based on TRIzol^®^ reagent (Ambion Thermo Fisher) with Direct-zol^™^ RNA Purification kit (Zymo Research) following manufacturer’s protocol. For the RNA extraction of encapsulated cells, the bioprinted scaffolds were enzymatically digested with collagenase IV to release the cells before applying TRIzol^®^ reagent. The RNA products were quantified using NanoDrop^™^ 2000 (Thermo Fisher Scientific). The reverse transcription was done using iScript^™^ cDNA Synthesis Kit (Bio-Rad) and the real time quantitative polymerase chain reaction (qPCR) was conducted using Luna^®^ Universal qPCR Master Mix (New England Biolabs). The primer information was enclosed in Supplementary Table 2.

### 2.8. RNA sequencing and transcriptomic analysis

For RNA sequencing (RNA-seq), hCjSCs from 3 healthy donors were expanded and labeled with GFP using lentiviral vectors before subjecting to bioprinting of the 3D pterygium model and co-culture. After 5–7 days of co-culture, the GFP-labeled hCjSCs were isolated from the bioprinted scaffolds by enzymatic digestion and fluorescence-activated cell sorting (FACS). Then, RNA samples were extracted as aforementioned and quantified using NanoDrop^™^ 2000 (ThermoFisher Scientific). The library preparation and RNA-seq were performed on Illumina platform by Novogene (Sacramento, CA). The transcriptomic data from patient-derived pterygium tissues and normal conjunctival tissues were derived from published datasets [49,50].

For the transcriptomic data analysis, the sequencing reads were filtered and trimmed with Trim Galore (version 0.4.1) followed by mapping to the human genome (GRCh38. p12) using HISAT2 [51]. The mouse genome (mm10) was also used to estimate the cross-mapping rates. Principle component analysis (PCA) was performed using DESeq package with the batch effect between different datasets filtered [52]. Differently expressed gene (DEG) analysis was performed using DESeq (Adjusted P-value<0.01). The DEGs with the same regulated expression pattern to the patient-derived pterygium tissues were defined as consistent DEGs. The protein-to-protein interaction (PPI) enrichment analysis based on DEGs was presented through Cytoscape [53]. The gene set enrichment analysis (GSEA) comparing the 3D pterygium model and 2D control was performed using GESA software (http://software.broadinstitute.org/gsea/downloads.jsp) [54]. The gene ontology (GO) enrichment analysis in this study was accomplished with Geneontology [55].

### 2.9. Imaging and image processing

Imaging in this study was conducted using Leica SP8 confocal microscope and Leica DMI 6000-B fluorescence microscope. Images were processed with LAS X and ImageJ.

### 2.10. Statistical analysis

Statistical analysis was performed using Microsoft Excel and GraphPad Prism. The data was presented as mean ± standard deviations with two-tailed Student’s t-test or one way ANOVA used to determine the significance. P-value was presented in the figures with asterisks (*: P < 0.05; **: P < 0.01; ***: P < 0.001.).

## 3. Results

### 3.1. In vitro expansion of primary hCjSCs

**Fig. 1.**
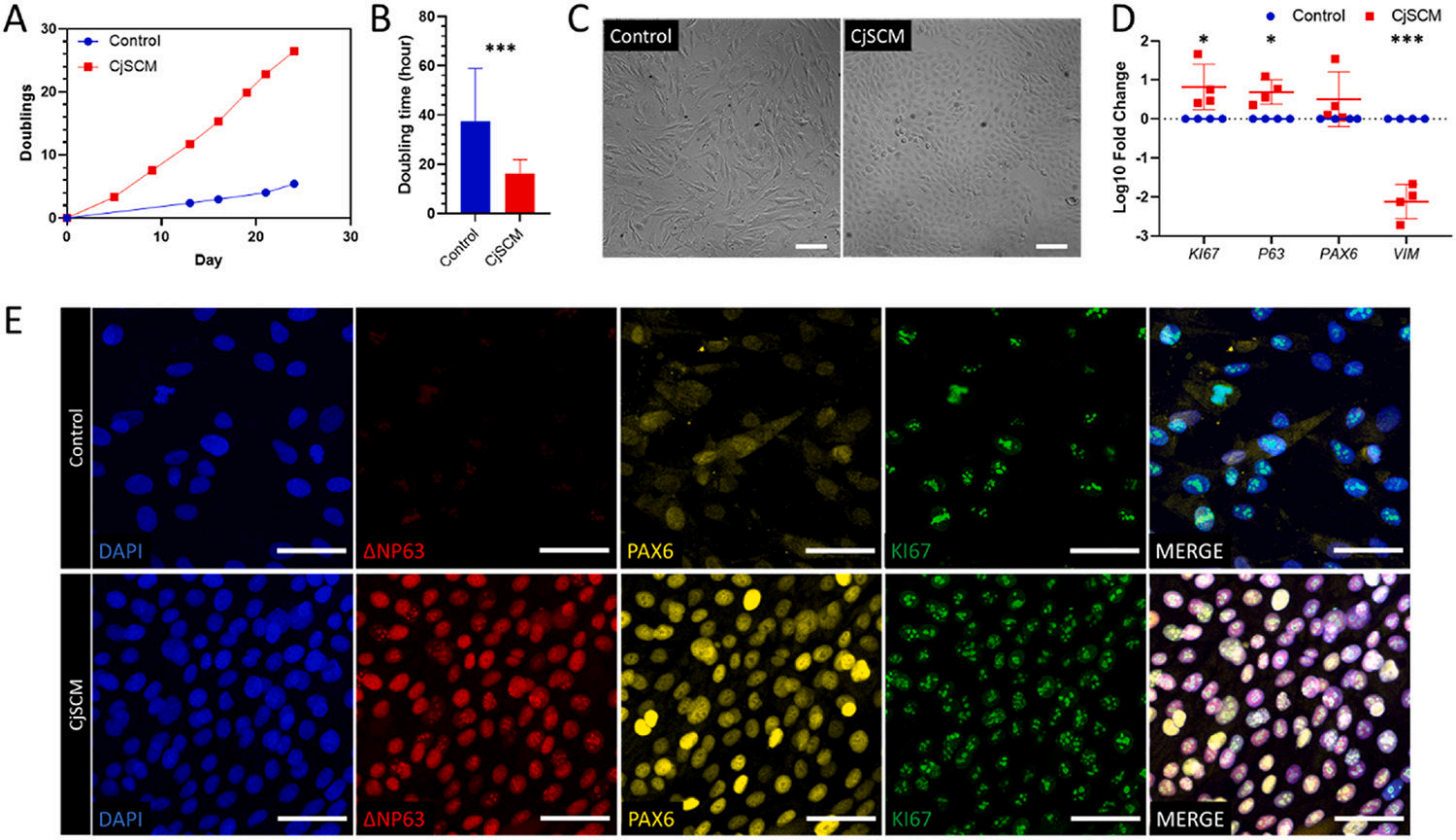
*In vitro* expansion of primary hCjSCs using CjSCM. (A) Representative cumulative quantification plot showed the cell doublings versus the culture time of the human primary conjunctival epithelial cells in culture with CjSCM or control medium. (B) Average cell doubling time of human conjunctival epithelial cells in culture with control medium and CjSCM from passage 1 to 8 (mean ± sd, n = 3; ***: P < 0.001). (C) Cell morphologies of nonconfluent primary human conjunctival epithelial cells cultured with CjSCM or control medium at passage 3. Scale bars: 100 μm. (D) Real time qPCR showing the relative mRNA expression of *KI67* (proliferation), *P63* (stemness), *PAX6* (ocular lineage), *VIM* (mesenchymal lineage) in the cells expanded in CjSCM or control medium (mean ± sd, n = 4, *: P < 0.05, ***: P < 0.001.). (E) Immunofluorescence staining of ΔNP63, PAX6 and KI67 on hCjSCs expanded in CjSCM or control medium at passage 3. Scale bars: 50 μm.

The hCjSCs are one of the predominant stem cells on the ocular surface with high value in clinical applications, but the *in vitro* expansion has been a challenge [39,40,43]. We have previously reported the feeder-free culture of primary rabbit CjSCs adopting a cocktail of small molecules that inhibit transforming growth factor-beta (TGF-β) signaling, bone morphogenetic proteins (BMP) signaling, and Rho-associated protein kinase (ROCK) signaling and selectively expanded CjSC population in primary culture [44]. Given the promising results of rabbit cells, we first validated the expansion efficacy of CjSCM on primary hCjSCs isolated from donor tissues. As shown in accumulative quantification of cell doublings, compared to the cells cultured in the control medium without inhibitor cocktail, those cultured in CjSCM exhibited faster dividing, a shorter cell doubling time and higher replicative potency (Fig. 1A and B, Supplementary Figure S1A). As for cell morphology, the cells expanded with CjSCM showed more compacted, cuboidal, and uniform morphology whereas the control cells were elongated, spindle-shaped, and of various sizes (Fig. 1C). In addition, as measured with real time qPCR, the mRNA expression of epithelial stem cell marker, *P63*, and proliferation marker, *KI67*, were significantly upregulated in the CjSCM group, while the expression of the mesenchymal marker vitronectin (*VIM*) was significantly downregulated compared with control (Fig. 1D). The immunofluorescence staining of stem cell markers (ΔNP63, P63, ABCG2, KRT14), lineage markers (PAX6, E-cadherin (ECAD)), and proliferation marker KI67 indicated the predominant presence of hCjSCs in CjSCM condition (Fig. 1E, Supplementary Figure S1B). To validate the stem cell potency, we differentiated the expanded hCjSCs into goblet cells. After 7 days of differentiation, the generation of conjunctival goblet cells was confirmed by protein expression of mucin 1 (MUC1), mucin 5AC (MUC5AC), and mucin 16 (MUC16) (Supplementary Figure S1C). These results collectively demonstrated that our CjSCM culture system could efficiently expand primary hCjSCs *in vitro* with high homogeneity while promoting the stem cell phenotypes and preserving the differentiation potency.

### 3.2. DLP-based 3D bioprinting of hCjSCs

**Fig. 2.**
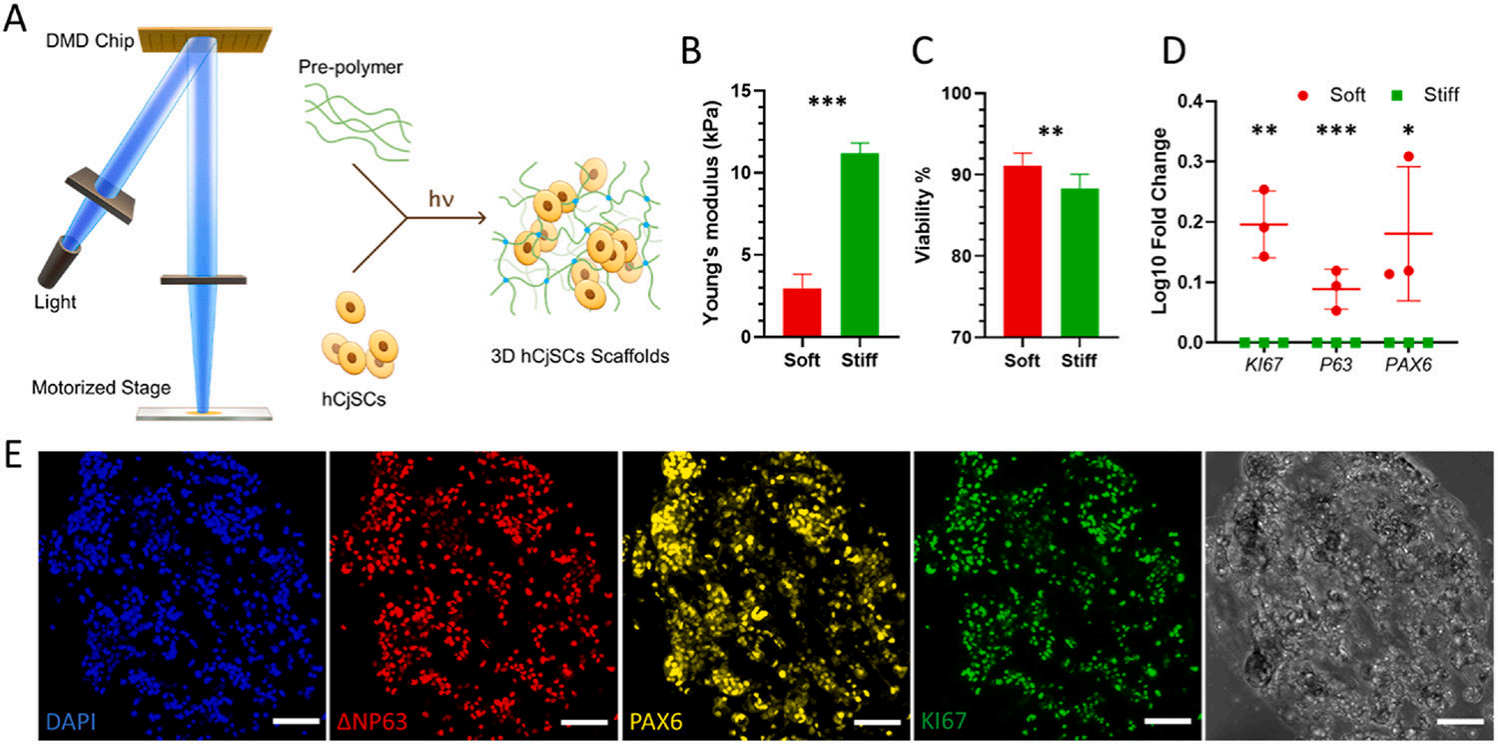
DLP-based 3D bioprinting of hydrogel scaffolds supporting the stemness and functionality of the encapsulated hCjSCs. (A) Schematics of the DLP bioprinter setup and the photopolymerization process to fabricate hydrogel scaffolds encapsulating hCjSCs. (B) Compressive modulus of the hCjSCs encapsulated in soft and stiff bioprinted scaffolds (mean ± sd, n = 3). (C) The ratio of PI-negative population measured with flow cytometry representing the percentage of viable cells in soft and stiff bioprinted scaffolds cultured for 5 days (mean ± sd, n = 3). (D) Real time qPCR showing the relative mRNA expression of *KI67, P63* and *PAX6* of hCjSCs in 2D culture condition (2D control) or 3D hydrogel scaffolds with different stiffness (mean ± sd, n = 3, *: P < 0.05, **: P < 0.01, ***: P < 0.001.). (E) Representative immunofluorescence staining and corresponding bright field images of bioprinted hydrogel scaffolds encapsulating hCjSCs after 2 days of culture expressing ΔNP63, PAX6 and KI67. Scale bars: 100 μm.

To support the application of hCjSCs in disease modeling, we next explored 3D bioprinting of hCjSCs. With the rapid and scalable process, high fabrication resolution and versatile material choice, DLP-based 3D bioprinting has been used in fabricating hydrogel scaffolds encapsulating various types of human stem cells for disease modeling and therapeutic purposes [29,33,46,47]. For the DLP-based bioprinting, hCjSCs were mixed with a prepolymer solution to form the bioink and photopolymerized to fabricate the 3D hydrogel scaffolds (Fig. 2A). GelMA was adopted as a bioink material because of its excellent cell binding capacity and has been successfully used for encapsulating multiple cell types, including rabbit CjSCs [29,44,56]. The extracellular matrix (ECM) stiffness has been shown to regulate the essential function and behavior of stem cells [57,58]. To optimize the encapsulation of hCjSCs, GelMA cylinders (diameter: 500 μm; thickness: 500 μm) encapsulating hCjSCs in soft (2.98 ± 0.85 kPa) and stiff (11.20 ± 0.62 kPa) condition were bioprinted and subjected to tissue culture (Fig. 2B). Flow cytometry with PI staining showed that both conditions had over 85% cell viability (Fig. 2C). Notably, the cell viability was significantly higher in soft condition (Fig. 2C). The high cell viability of encapsulated cells was also confirmed by LIVE/DEAD^®^ staining (Supplementary Figure S2A). In addition, real time qPCR indicated that the mRNA expression of *KI67, P63*, and *PAX6* were significantly higher in the soft condition (Fig. 2D). Thus, we adopted the soft printing condition for the following experiments. The stem cell identity of the hCjSCs in the bioprinted 3D scaffolds was retained (Fig. 2E, Supplementary Figure 2B). To test the cell functionality, we conducted 3D differentiation of hCjSCs encapsulated in the bioprinted scaffolds and found the expression of characteristic mucins 7 days later (Supplementary Figure S2C). Together, using the DLP-based 3D bioprinting, we fabricated microscale GelMA hydrogel scaffolds encapsulating hCjSCs while preserving the cell viability, stemness and functionality.

### 3.3. 3D bioprinted multicellular pterygium model

**Fig. 3.**
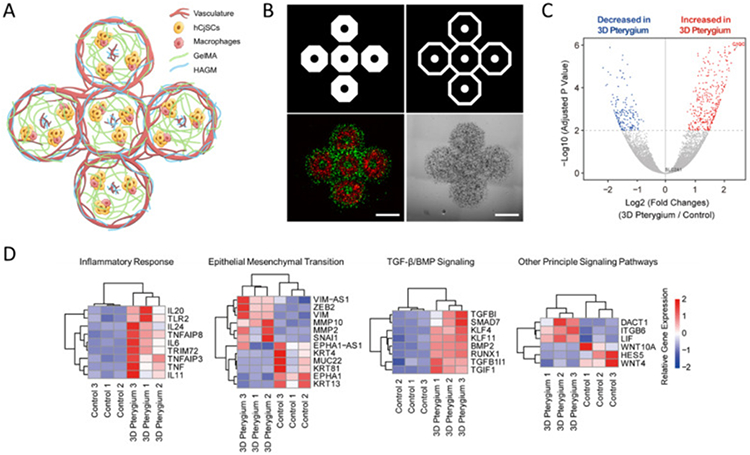
DLP-based 3D bioprinting of multicellular pterygium model with distinct transcriptomic profiles. (A) Illustration of the bioprinted multicellular 3D pterygium model. (B) Representative images of the 3D pterygium model. Red: hCjSCs and macrophages; green: HUVECs and fibroblasts. Scale bars: 1 mm. (C) Volcano plot of global transcriptomic landscape comparing the bioprinted 3D pterygium model with the 2D control. The *x*-axis represents log2 transformed fold changes, and the *y*-axis shows the −log10 transformed *p*-value adjusted for multiple test correction (*n* = 3 per condition). (D) Heatmap of representative DEGs correlated to inflammatory response, epithelial mesenchymal transition, TGF-β/BMP signaling, and other principal signaling pathways in the 3D pterygium model versus the 2D control. Scale bars represent relative gene expression (log2 fold changes).

With the background of extensive chronic inflammation, angiogenesis and infiltration of immune cells dominate in the pterygium pathology [14,17,48,59,60]. Some studies also implicated the involvement of stem cells in pterygium [14,61]. Existing pterygium disease models employed subconjunctival delivery of patient-derived pterygium epithelial cells or fibroblasts to induce immune response and neovascularization, but little attention has been paid to developing an *in vitro* model with a multicellular microenvironment [17,18,20]. Taking advantage of 3D bioprinting of hCjSCs, we developed a 3D bioprinted pterygium model with conceptualized patterns containing hCjSCs, macrophages, vascular endothelial cells, and fibroblasts to recapitulate the pathological state of pterygium (Fig. 3A). The model contained two layers: the first layer containing hCjSCs and THP-1-derived macrophages recapitulated the infiltration of immune cells during the inflammation response; the second layer with HUVECs and 10T1/2s cells represented the angiogenesis around and inside the pterygium tissue. Different populations of cells were bioprinted and subjected to co-culture for 5–7 days (Fig. 3B). Immunofluorescence staining showed the presence of vascular markers, CD31 and vascular endothelial cadherin (VE-CAD) after 6 days of co-culture, suggesting the formation of microvasculature in the bioprinted 3D pterygium model (Supplementary Figure S3A).

### 3.4. 3D pterygium model displayed distinct transcriptomic profiles compared with 2D culture

To comprehensively characterize the 3D pterygium model, we bioprinted the models with hCjSCs from three normal individual donors and performed global transcriptomic profiling with RNA-seq. The hCjSCs cultured on collagen-coated surface were used as control, and the hCjSCs that were extracted from 3D bioprinted scaffolds with only hCjSCs were used as 3D control. Prior to the analysis, the sequencing reads were mapped with both the human and mouse genome database and confirmed the absence of mouse RNA fragments (Supplementary Figure S3B).

PCA showed a drastic transcriptomic difference between the 3D pterygium model and the controls (Supplementary Figure S3C). 590 DEGs were identified in 3D pterygium compared to the 2D condition, among which 420 genes were significantly upregulated in the 3D pterygium model, whereas 170 genes were downregulated (Fig. 3C, Supplementary Figure S3D). We also found that 311 genes were significantly upregulated while 555 genes were downregulated in the 3D pterygium compared to the 3D control (Supplementary Figure S4A). Based on the DEG analysis comparing with the 2D control, the 3D pterygium models showed consistent upregulation of genes correlated to interleukin cascade, tumor necrosis factor (TNF) signaling, and other inflammatory responses (Fig. 3D). We also noticed the upregulation of mesenchymal markers and epithelial-mesenchymal transition (EMT) related genes, along with the downregulation of epithelial markers (Fig. 3D). In addition, principle signaling pathways altered in the 3D pterygium model were identified, indicating the upregulation of TGF-β/BMP signaling, which is a regulator of EMT and significantly regulates the recurrence of pterygium (Fig. 3D) [62]. These results underlined that the encapsulated hCjSCs in the bioprinted 3D pterygium model were regulated by the synthetic microenvironment and underwent inflammatory responses and EMT [63-65]. Furthermore, we have found that the expression of multiple epigenetic regulators was upregulated in the 3D pterygium model, including DNA methyltransferase DNMT3B, lysine demethylase KDM6B, and histone deacetylase HDAC5 (Supplementary Figure S4B). As epigenetic regulation has been shown to involve in both pterygium pathogenesis and 3D tissue microenvironment development, these results could indicate the recapitulation of epigenetic activities in the 3D pterygium model [66] [–] [68].

### 3.5. 3D pterygium model exhibited transcriptomic signatures of pterygium

**Fig. 4.**
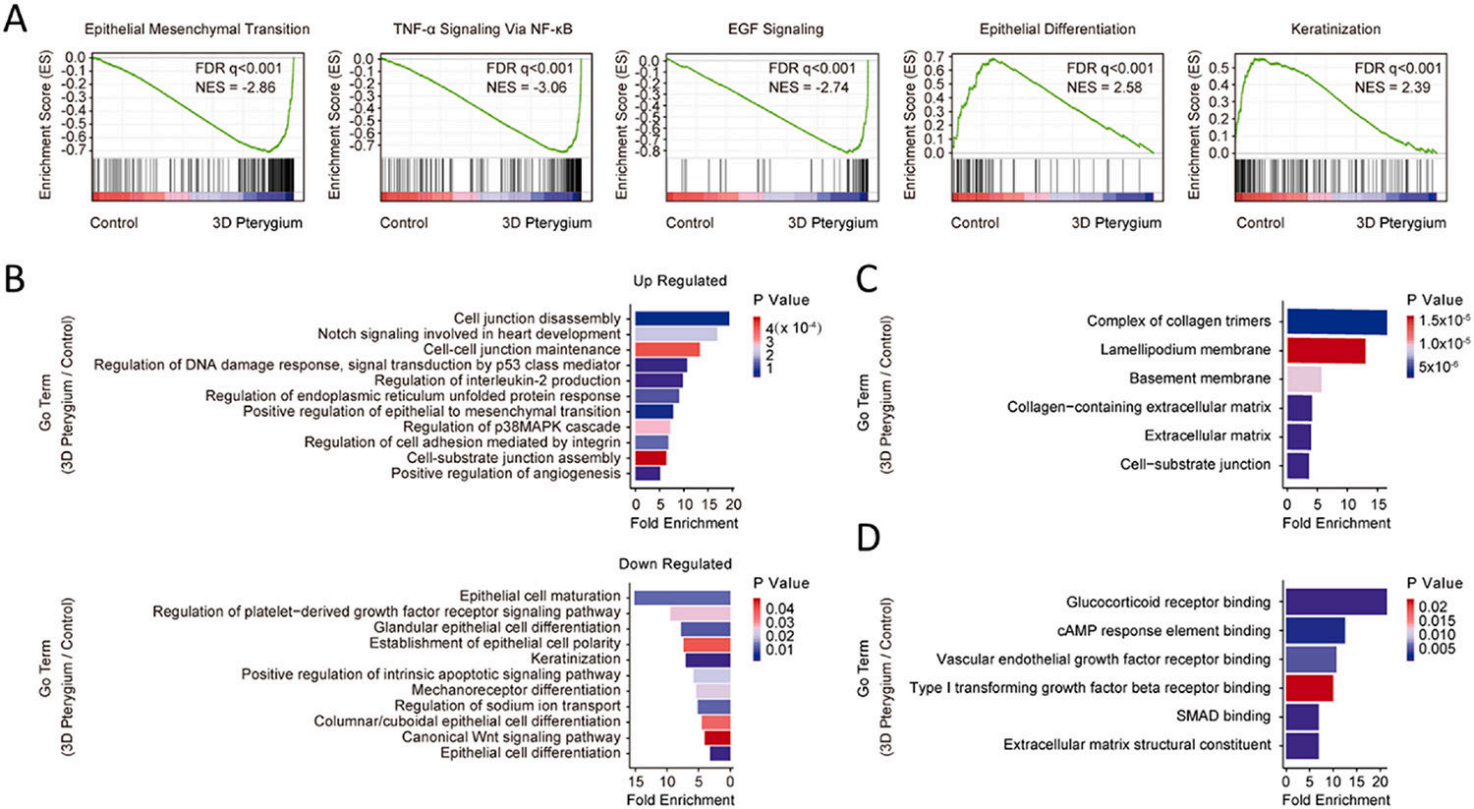
GSEA and GO analysis revealed the pterygium-related pathological features in the 3D pterygium model. (A) Representative GSEA results comparing the 3D pterygium model with the control. FDR: false discovery rate, NES: normalized enrichment score. (B) GO terms enriched in hCjSCs cultured in the 3D pterygium model versus 2D control. (C) Selected upregulated GO terms from the cellular component domain in the 3D pterygium model. (D) Selected upregulated GO terms from the molecular function domain in the 3D pterygium model.

**Fig. 5.**
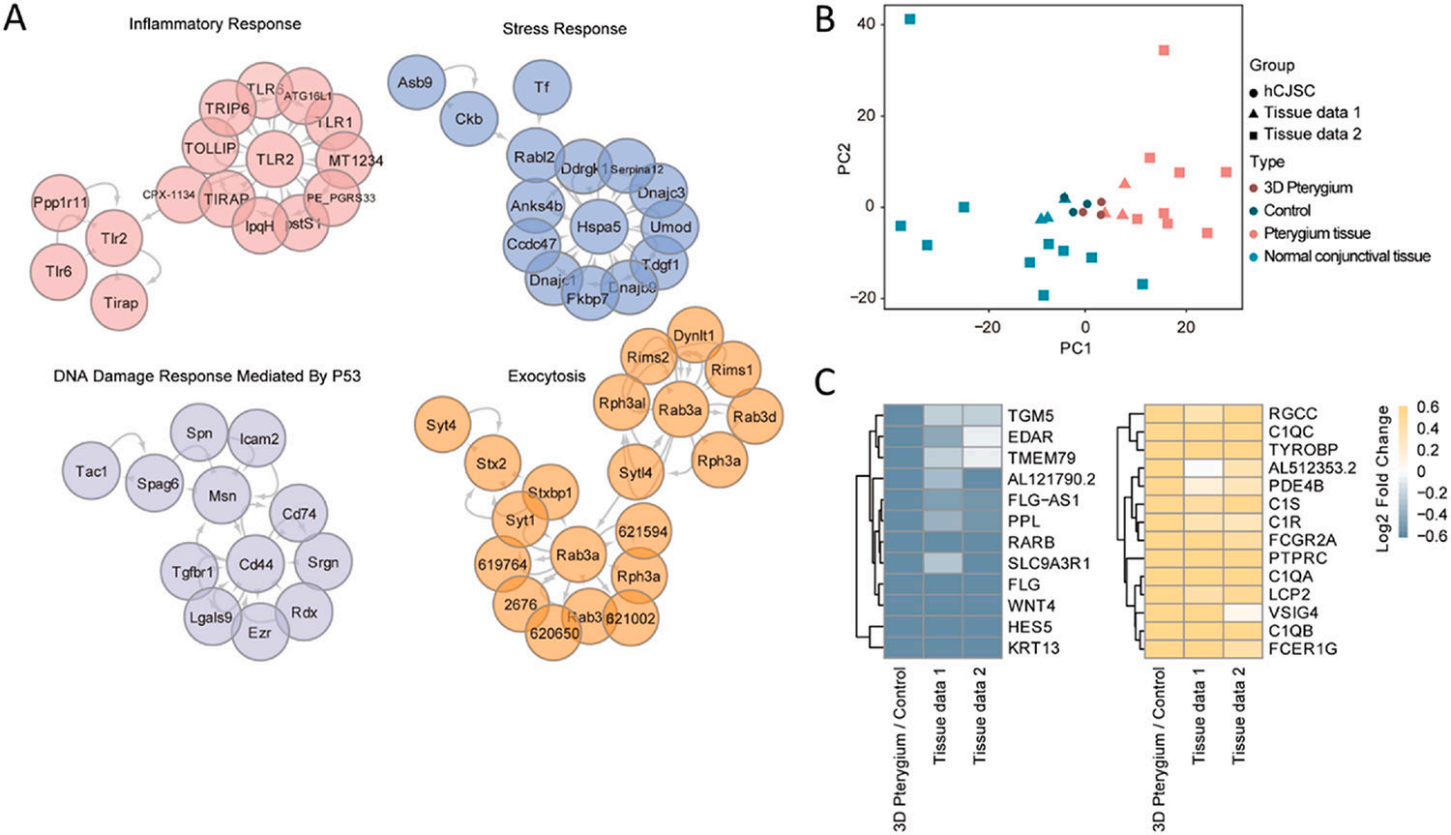
Transcriptome profiles of 3D pterygium model resemble a patient-derived pterygium tissue. (A) PPI enrichment analysis based on the DEGs between the 3D pterygium model and the control. (B) PCA of the global transcriptomic profiles of the hCjSCs from the bioprinted model (3D pterygium) and 2D culture (Control), and human tissues from healthy individuals (normal conjunctival tissue) and pterygium patients (Pterygium tissue). (C) Heatmap of consistent DEGs correlated to activation of immune response and epithelial cell differentiation. Tissue data 1 (X. Liu et al.) and tissue data 2 (Y. Chen et al.) represent human tissue data from two independent studies. Scale bar represents normalized fold change.

To further understand the molecular features of the 3D pterygium model, we performed GSEA and GO enrichment analysis. GSEA revealed that the 3D pterygium model expressed enriched gene hallmarks of TNF-α/NF-κB signaling, EMT, and EGF signaling, while the controls showed enrichment involved in epithelial differentiation and keratinization (Fig. 4A, Supplementary Figure S4C). GO enrichment analysis showed the overrepresented GO terms were correlated to the organization of cell-cell and cell-substrate junction, EMT, Notch signaling, DNA damage response, endoplasmic reticulum (ER) unfolded protein response (UPR), interleukin production, and angiogenesis regulation in the 3D pterygium model, while GO terms correlated to epithelial cell differentiation, keratinization, and canonical Wnt signaling were downregulated (Fig. 4B). The top up-regulated terms from the cellular component domain highlighted the ECM organization and the cell-cell, cell-substrate interaction (Fig. 4C). The key molecular function terms involved in glucocorticoid receptor signaling, vascular endothelial growth factor (VEGF) signaling, and TGF-β signaling (Fig. 4D). In comparison with the 3D control, the 3D pterygium model showed upregulated GO terms correlated to platelet-derived growth factor receptor (PDGFR) signaling pathway, EMT, angiogenesis, inflammatory response and cell junction organization (Supplementary Figure S4D). Consistently, PPI enrichment analysis highlighted the protein networks associated with inflammatory response, stress response, DNA damage response, and exocytosis in the 3D pterygium model (Fig. 5A). To investigate to what extent the 3D pterygium model could imitate the molecular features of pterygium, we combined the RNA-seq data of normal human conjunctiva and patient-derived pterygium sample from previously published datasets for integrated analysis [49,50]. PCA analysis distinguishes the patient-derived pterygium tissues from the normal conjunctival tissues from healthy donors (Fig. 5B). In addition, the 3D pterygium model exhibits similar transcriptional pattern to patient-derived pterygium tissues but with higher homogeneity (Fig. 5B). To investigate the common gene regulation network between the 3D pterygium model and patient-derived pterygium tissues, we focused on the DEGs of the 3D model that showed consistent expression pattern in the patient-derived pterygium. Totally, 189 consistent upregulated DEGs and 81 consistent downregulated DEGs were identified (Supplementary Table 3). GO enrichment analysis on consistent DEGs comparing different datasets revealed that the 3D pterygium model had upregulation GO terms consistent with the pterygium tissues correlated to activation of immune response, the regulation of cell-cell junctions and cell-substrate junctions, EMT, VEGF production, integrin-mediated signaling pathways, non-canonical Wnt signaling (planar cell polarity), and TGF-β/SMAD signaling (Fig. 5C, Supplementary Figure S4E). These results indicated that the bioprinted 3D pterygium model was able to recapitulate the disease microenvironment of pterygium and transition healthy hCjSCs into the pterygium-relevant pathological state.

## 4. Conclusion

Pterygium is a pathological conjunctival overgrowth with chronic inflammation and angiogenesis that could result in blindness [7,8,16]. Effective and reproducible disease models are needed to decipher the pathogenesis and explore new therapeutic approaches for pterygium [18,19]. Here, based on *in vitro* expansion and DLP-based 3D bioprinting of hCjSCs, we developed an *in vitro* multicellular pterygium disease model. The bioprinted pterygium model consisted of hCjSCs from healthy donors, macrophages, HUVECs, and fibroblasts mimicking the multicellular pterygium microenvironment. By performing global transcriptomic analysis with RNA-seq, we found that the hCjSCs in the bioprinted 3D model exhibited pathological features highlighting inflammatory response and EMT. Further comparative analysis with published data of patient-derived pterygium tissues confirmed the presence of pterygium signatures in our bioprinted 3D pterygium model.

Despite the clinical need of hCjSCs, protocols for developing effective hCjSC models have not been publicly reported in full [37,39,42]. Consistent with other reported culture of human epithelial stem cells and our previous report on rabbit CjSCs, CjSCM with the inhibition on TGF-β signaling and BMP signaling, as well as the ROCK signaling, was able to support the efficient *in vitro* expansion of hCjSCs while maintaining the stemness and differentiation potency [44,69,70]. As we generated large amount of cells from a small amount of starting materials for the experiment, our culture method could combine with impression cytology for a future clinical study [71]. In addition, with the DLP-based 3D bioprinting, we fabricated GelMA hydrogel scaffolds supporting the viability and stem cell properties of the encapsulated hCjSCs. The rapid, scalable, reproducible fabrication with DLP-based 3D bioprinting made this model highly valuable and clinically translatable for personalized medicine [72]. The flexible pattern design also enabled convenient modification on the models to adapt different biomedical applications in the future.

3D engineered models with control in geometry, cell distribution, and ECM composition were shown to better mimic the physiological or pathological microenvironment compared to the 2D cell monolayer and had higher scalability and reproducibility over animal models [25,73, 74]. To recapitulate the pterygium microenvironment, we integrated hCjSCs with immune cells and vascular cells in the bioprinting to develop a 3D pterygium model and performed RNA-seq to evaluate the model [12,60]. Vast differences in gene expression were found in DEG analysis comparing hCjSCs from the 3D pterygium model with the 2D and 3D control, indicating the bioprinted multicellular 3D microenvironment significantly altered the state of encapsulated cells. The GSEA and GO enrichment analysis indicated that the hCjSCs in the 3D pterygium model were under ER stress and DNA damage, which were potentially induced by the inflammatory stimulus through TNF-α/NF-κB signaling and interleukin cascade [75-77]. As a result, the cells underwent EMT that was potentially mediated by integrin signaling, TGF-β/SMAD signaling, and Notch signaling [63,78] [–] [81]. In addition, GSEA identified the activation of EGF signaling in the 3D pterygium model, underlining the crosstalk between epithelial cells and macrophages [82,83]. Notably, by comparing our data with the transcriptomic signatures identified in the patient-derived samples, the bioprinted 3D pterygium model was grouped into the pterygium tissues whereas the 2D control was classified into healthy conjunctival tissues, which further confirmed the pathological changes of healthy hCjSCs in the bioprinted model [49,50]. Moreover, the key events and signaling pathways that were highlighted by the transcriptomic analysis are potential targets for developing pharmaceutical treatment of pterygium.

In conclusion, through 3D bioprinting of hCjSCs, we have developed a bioprinted 3D pterygium model presenting the multicellular microenvironment and transcriptomic signatures of pterygium. This is the first reported 3D *in vitro* disease model for pterygium recapitulating pathological features consistent with patient-derived pterygium tissues. Supported by the DLP-based 3D bioprinting technology, this model can potentially support therapeutic development and high-throughput drug screening, as well as the disease mechanism study of pterygium.

## Supplementary Material

suppl

## Data Availability

The raw data for the RNA-seq analysis is available on NCBI Gene Expression Omnibus (GEO) with the accession number of GSE180343. The other raw/processed data required to reproduce these findings can be shared by the authors upon request.

## Abbreviations

CjSC: Conjunctival stem cells
DLP: Digital light processing
RNA-seq: RNA sequencing
GelMA: gelatin methacryloyl
HAGM: hyaluronic acid glycidyl methacrylate
