# Supplementary material for: Rapid 3D bioprinting of a multicellular model recapitulating pterygium microenvironment
Source: Biomaterials. Author manuscript; Available in PMC 2023 May 5. (PMC10162446; doi:10.1016/j.biomaterials.2022.121391)
Supplement: suppl [file NIHMS1891775-supplement-suppl.zip › 1-s2.0-S0142961222000308-mmc5.docx]

**Supplementary Materials**

**Rapid 3D Bioprinting of a Multicellular Model Recapitulating Pterygium Microenvironment**

*Zheng Zhong^a,1^, Jing Wang ^b,1^, Jing Tian^a,1^, Xiaoqian Deng ^b^, Alis Balayan ^a,c^, Yazhi Sun^a^, Yi Xiang^a^, Jiaao Guan ^a^, Jacob Schimelman^a^, Henry Hwang^a^, Shangting You*^a^*, Xiaokang Wu ^c^, Chao Ma^d^, Xiaoao Shi^a^, Emmie Yao^a^, Sophie X. Deng^d^, Shaochen Chen^a*^*


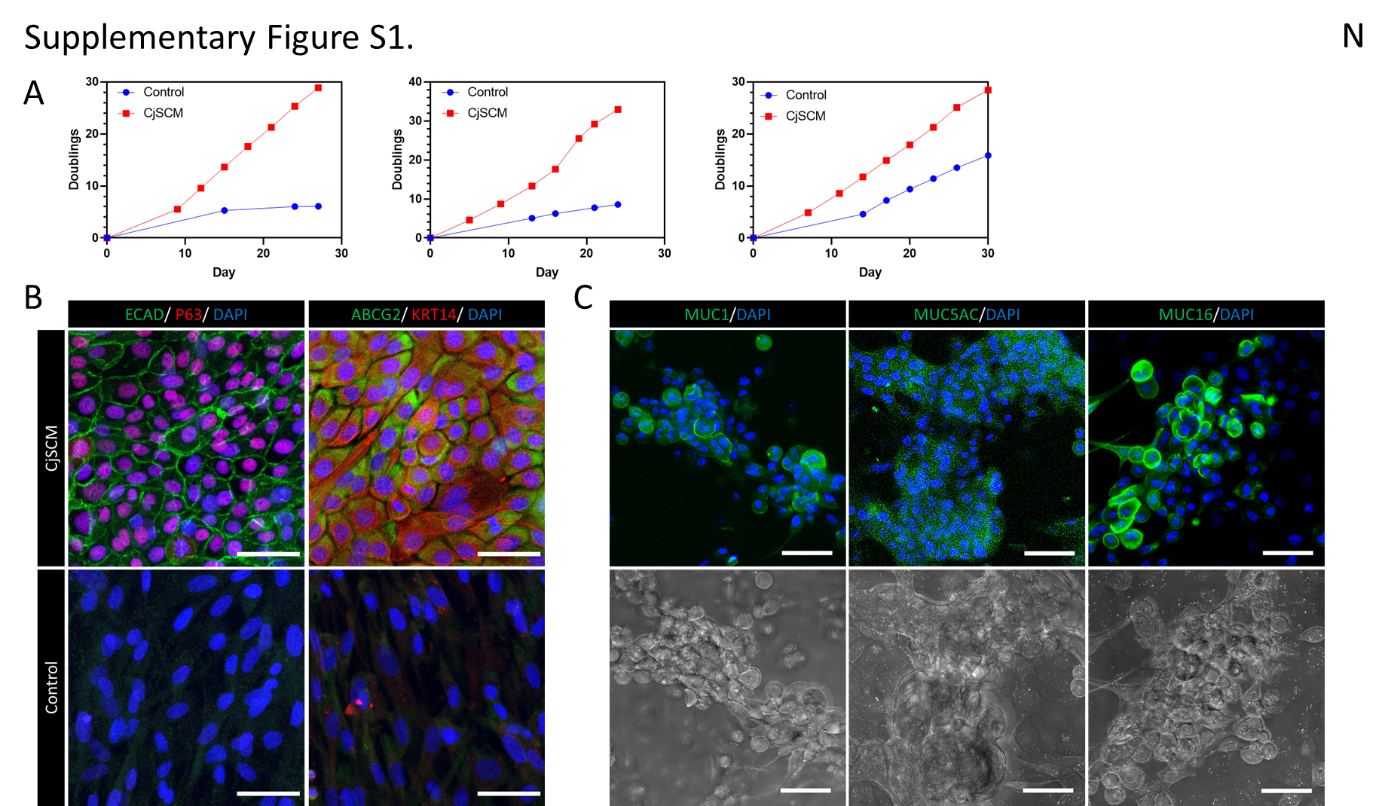


Supplementary Figure S1. (A) Cumulative quantification plots of the cell doublings versus the culture time of primary conjunctival epithelial cells from different individual donors in culture with CjSCM or control medium. (B) Immunofluorescence staining of ECAD/P63 and ABCG2/KRT14 on hCjSCs expanded in CjSCM or control medium at passage 3. Scale bars: 50 μm. (C) Immunofluorescence staining of MUC1, MUC5AC and MUC16, and the corresponding bright field images on the conjunctival goblet cells differentiated from hCjSCs. Scale bars: 50 μm.


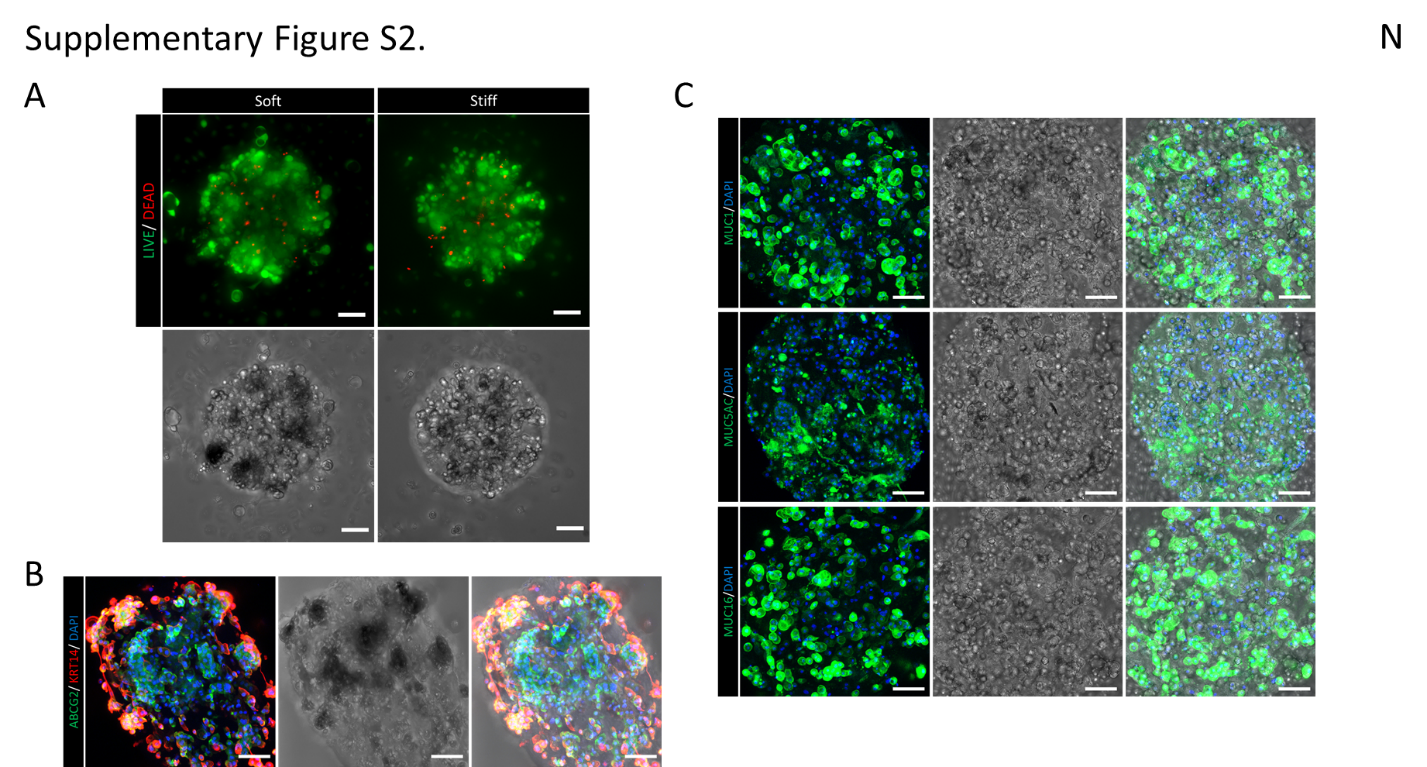


Supplementary Figure S2. (A) Live/Dead® staining was performed on the hydrogel scaffolds encapsulating hCjSCs to compare cell viability in soft and stiff scaffolds after 2 days in culture. Scale bars: 100 μm. (B) Immunofluorescence staining of ABCG2 and KRT14 on bioprinted hydrogel scaffolds encapsulating hCjSCs after 2 days in culture. Scale bars: 100 μm. (C) Immunofluorescence staining of MUC1, MUC5AC and MUC16 on hydrogel scaffolds encapsulating hCjSCs after 7 days of conjunctival goblet cell differentiation. Scale bars: 100 μm.


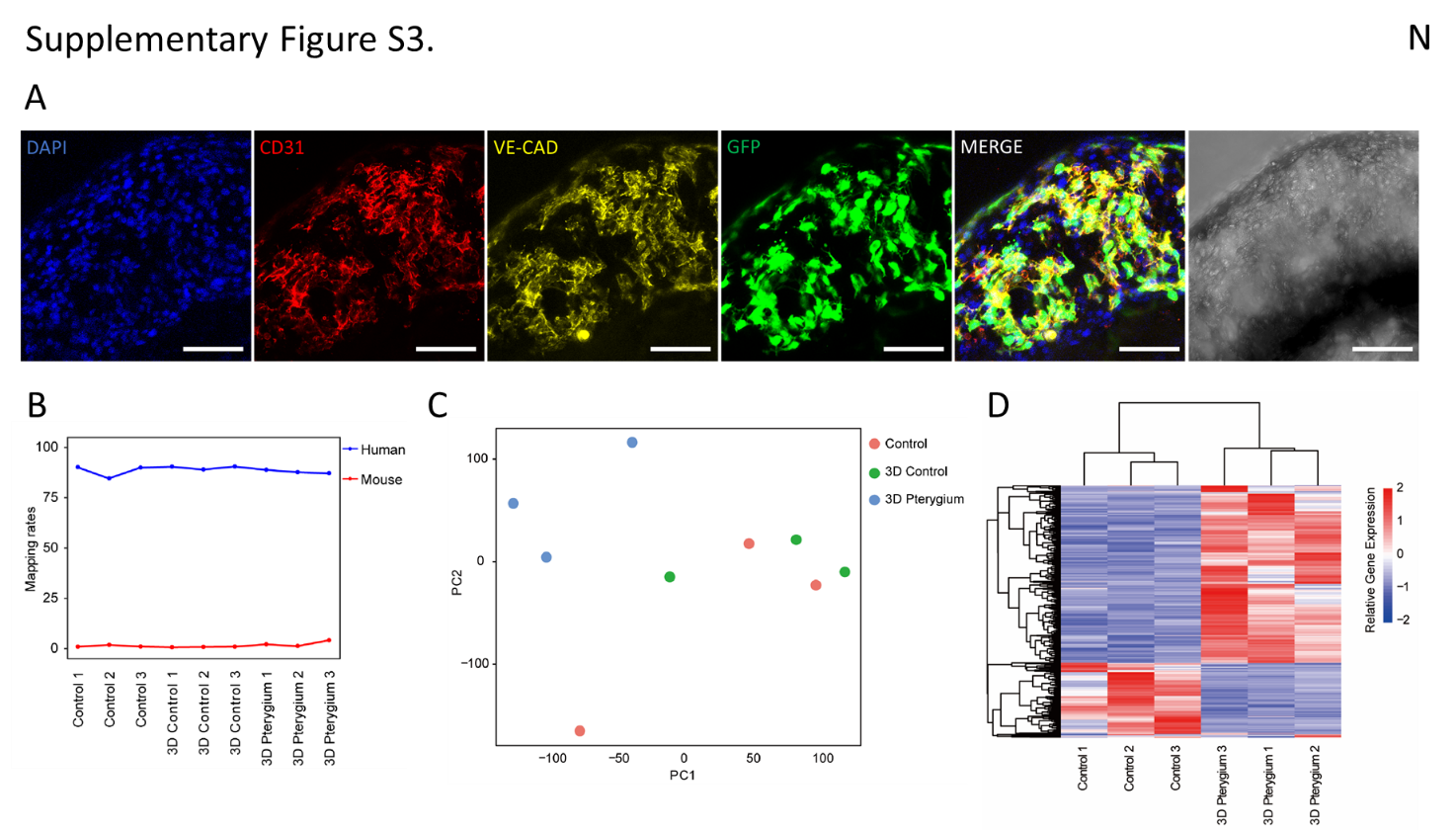


Supplementary Figure S3. (A) Representative immunofluorescent image of the micro-vasculature with the staining of CD31 and VE-CAD in the bioprinted 3D pterygium model after 6 days of co-culture. Scale bars: 100 μm. (B) The mapping rates of the sequencing data with the human genome and the mouse genome. (C) PCA result of the global transcriptomic profiles of the control, the 3D control and the 3D pterygium model. (D) Heatmap showing the unsupervised hierarchical clustering based on the DEGs between the 3D pterygium model and the control. Scale bars represent relative gene expression (log2 fold changes).


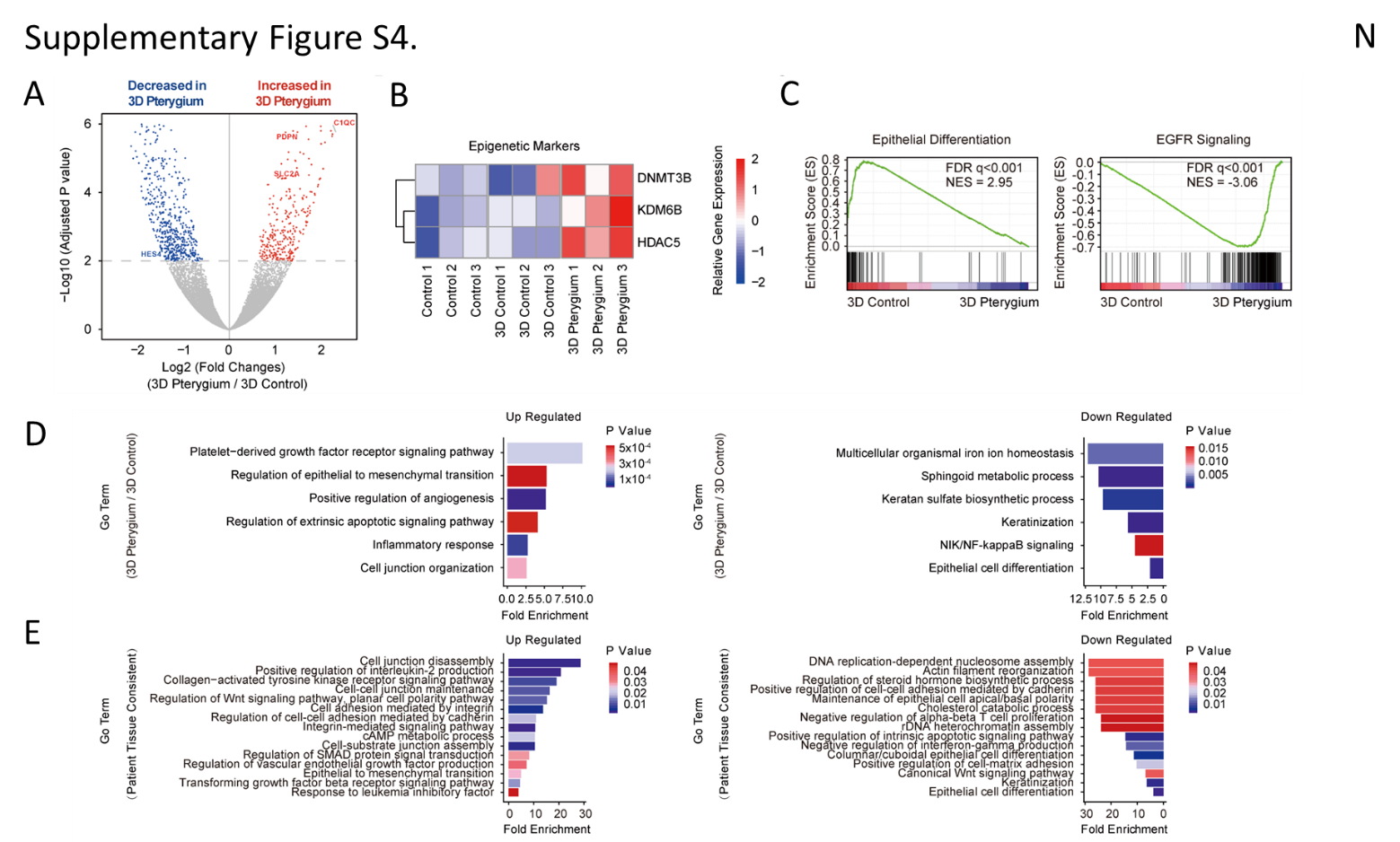


Supplementary Figure S4. (A) Volcano plot of global transcriptomic landscape comparing the 3D pterygium model with the 3D control. (B) Representative epigenetic markers in DEGs. Scale bars represent relative gene expression (log2 fold changes). (C) Representative GSEA showing the enrichment of EGFR signaling in the 3D pterygium compared to the 3D control, and epithelial differentiation in the 3D control compared to the 3D pterygium. (D) GO terms enriched in hCjSCs cultured in the 3D pterygium model versus 3D control. (E) GO enrichment analysis on the consistent DEGs in 3D pterygium model and human patient samples.
